# Psychosocial and Somatosensory Factors in Women with Chronic Migraine and Painful Temporomandibular Disorders

**DOI:** 10.1155/2016/3945673

**Published:** 2016-10-13

**Authors:** Alfonso Gil-Martínez, Mónica Grande-Alonso, Roy La Touche, Manuel Lara-Lara, Almudena López-López, Josué Fernández-Carnero

**Affiliations:** ^1^Foundation for Biomedical Research, La Paz University Hospital, IDIPAZ, Po de la Castellana 261, 28046 Madrid, Spain; ^2^Motion in Brains Research Group, Centro Superior de Estudios Universitarios La Salle, Universidad Autónoma de Madrid, C/La Salle 10, 28036 Madrid, Spain; ^3^Institute of Neuroscience and Craniofacial Pain (INDCRAN), C/Caños del Peral 11, 28013 Madrid, Spain; ^4^Department of Physiotherapy, Centro Superior de Estudios Universitarios La Salle, Universidad Autónoma de Madrid, C/La Salle 10, 28036 Madrid, Spain; ^5^Rey Juan Carlos University, Avda. Atenas S/N, Alcorcón, 28922 Madrid, Spain; ^6^Multidisciplinary Group on Pain Research and Management, Excellence Research Program URJC-Santander, Alcorcón, Madrid, Spain

## Abstract

*Introduction*. Psychosocial and somatosensory factors are involved in the pathophysiology of chronic migraine (CM) and chronic temporomandibular disorders (TMD).* Objective*. To compare and assess the relationship between pain catastrophizing and kinesiophobia in patients with CM or chronic TMD.* Method*. Cross-sectional study of 20 women with CM, 19 with chronic TMD, and 20 healthy volunteers. Pain catastrophizing and kinesiophobia were assessed. The level of education, pain intensity, and magnitude of temporal summation of stimuli in the masseter (STM) and tibialis (STT) muscles were also evaluated.* Results*. There were significant differences between the CM and chronic TMD groups, compared with the group of asymptomatic subjects, for all variables (*p* < .05) except kinesiophobia when comparing patients with CM and healthy women. Moderate correlations between kinesiophobia and catastrophizing (*r* = 0.46; *p* < .01) were obtained, and the strongest association was between kinesiophobia and magnification (*r* = 0.52; *p* < .01). The strongest associations among physical variables were found between the STM on both sides (*r* = 0.93; *p* < .01) and between the left and right STT (*r* = 0.76; *p* < .01).* Conclusion*. No differences were observed in pain catastrophizing and kinesiophobia between women with CM and with chronic TMD. Women with CM or chronic TMD showed higher levels of pain catastrophizing than asymptomatic subjects.

## 1. Background

Pain is defined as an unpleasant sensory and emotional experience associated with actual or potential tissue damage or is described in terms of such damage [1]. According to Medical Subject Headings, pain is considered chronic when it is an aching sensation that persists for more than a few months. It might or might not be associated with trauma or disease and can persist after the initial injury has healed. Its localization, character, and timing are more vague than with acute pain.

Headaches and orofacial pain are a problem that affects a significant percentage of the population [1, 2]. Primary headaches are grouped within the International Classification of Headache Disorders (ICHD-3) and include chronic migraine (CM) [3]. This classification has been shown to be accurate in differentiating between migraines and tension headaches [4]. Four percent of the adult population experiences some type of chronic headache, with a higher incidence in women between 20 and 50 years of age [5]. Expanding our knowledge in this population is therefore especially important. In Spain, the prevalence of CM is 2.4% [6–8] and represents the most common clinical diagnosis in terms of daily headaches in the specialized headache units [9]. CM is highly disabling. Stovner et al. recently concluded that CM was more disabling than episodic migraine in the population regarding missed days of work, household chores, nonwork activity, and days with substantially reduced productivity over a 3-month period [5].

Orofacial pain is defined as that which includes various manifestations in the face or oral cavity and includes numerous conditions, such as temporomandibular disorders (TMDs) [10]. The prevalence of TMDs has been estimated between 3.7% and 12% and is 3 to 5 times more common in women. Approximately 3%–7% of patients have been treated for this disease at some point [11]. These disorders are characterized by their multifactorial etiology because of their related functional, structural, and psychological factors [12–14]. One of the most widely used classifications for the diagnosis, assessment, and categorization of TMDs is the Research Diagnostic Criteria for TMDs (RDC/TMD). This classification is based on a biobehavioral pain model with 2 axes: the physical axis (axis I) and the axis based on psychosocial and disability factors (axis II). Axis I includes painful myofascial disorders, disc displacements, and degenerative joint disorders [15, 16]. Although the pathophysiology of TMD is still unknown, a number of studies have identified the importance of the trigeminocervical nucleus as partly responsible for the pain modulation and amplification in this region [17].

Numerous studies have confirmed the important role of psychological and societal variables in CM and chronic TMD [18, 19], as occurs in other types of chronic pain. Among the variables studied in regard to chronic pain, pain catastrophizing (defined as an excessive response by an individual to their pain) bears special relevance [10]. High levels of pain catastrophizing are related to a greater intensity of pain [20, 21] and have been proposed to be a key element in the processes of central sensitization [22]. Pain catastrophizing has also been revealed as an element of considerable importance in the processes of pain chronicity and is related to another key variable in this process: fear of movement or kinesiophobia, which has been proposed as a mediator between catastrophism and pain [23]. Previous studies have indicated that patients with chronic headaches have high levels of kinesiophobia, which increases their disability [24]. There is insufficient scientific evidence, however, to demonstrate a relationship between pain catastrophizing and kinesiophobia in patients with CM or with chronic TMD.

The scientific evidence has shown that a percentage of these patients are susceptible to developing a central sensitization process [25–27], which is defined as a neuronal signaling amplification in the central nervous system (CNS), resulting in an experience of pain that does not necessarily reflect the presence of an adverse peripheral stimulus [26].

A number of authors have used the magnitude of the temporal summation of stimuli as an indicator of a possible impairment in the processing of pain related to the presence of central sensitization in a number of diseases [28], as is the case with a number of chronic TMDs [29]. This repetition of nociceptive stimuli activates the glutamate receptors (N-methyl-D-aspartate) [30–32]. This phenomenon would explain how a higher ratio in the temporal summation of stimuli leads to greater central impairment of nociceptive processing, as well as producing changes in the somatosensory function of the periphery, medulla, and brain [33].

In terms of the relationship between educational level and health status, a low educational level has been associated with greater pain catastrophizing and depressive symptoms when faced with pain [34].

Thus, our study's primary hypothesis was that patients with a diagnosis of CM or chronic TMD would show no differences in the previously recorded variables regarding pathophysiology.

The primary objective of the study was to compare and assess the relationship between pain catastrophizing and a fear of movement in patients with CM or with chronic TMD. The secondary objective was to assess the association between the somatosensory and psychological variables in these patients.

## 2. Patients and Methods

A cross-sectional study was conducted at La Paz University Hospital (HULP), with the approval of its ethics committee (PI-1241). Data were collected between October 2013 and April 2014. We followed the international recommendations of the Strengthening the Reporting of Observational Studies in Epidemiology checklist [35] for strengthening observational studies.

The participants were recruited simultaneously through the Departments of Neurology or Maxillofacial Surgery of HULP and the Spanish Association of Patients with Headaches, who were diagnosed with CM by a neurologist. The study also included asymptomatic participants with sociodemographic characteristics similar to those of the patients with CM or with chronic TMD. The former acted as controls and were occasionally companions of the HULP patients.

Each patient of legal age was placed into one of the following groups according to their diagnosis: (a) diagnosis of CM (according to the ICHD-3rd ed.) or (b) diagnosis of muscle, joint, and/or combined chronic TMD according to the RDC/TMD classification.

The following exclusion criteria were considered for the patients: concomitance with systemic rheumatic disease (including fibromyalgia) or CNS disease; nonchronic headache (according to ICHD-3rd ed.) in the CM group; a combined diagnosis of CM and TMD of any type; recent trauma or recent surgery in the head, face, neck, or chest; and pregnancy. All the exclusion criteria were checked using the medical history and physician judgment.

To assess the fear of movement and pain-related fear, we used the Spanish-validated version of the Tampa Scale for Kinesiophobia, which has good psychometric properties [36]. The level of pain catastrophizing was assessed with the Spanish-validated version of the Pain Catastrophizing Scale (PCS), which consists of 13 items and which has been shown to have appropriate psychometric properties [37]. To assess the magnitude of the temporal summation, we used Von Frey filaments. In this test, the patients were placed in supine decubitus on the stretcher and a measurement was performed in the masseter and another in the tibialis anterior muscle. Initially, a single stimulus was performed on these points. The patient then assessed the pain intensity of the stimulus using a visual analogue scale. Ten rhythmic stimuli were then performed on the same point guided by a metronome at 60 bpm. The pain intensity was once again assessed using the same scale [38]. To calculate the definitive value, we used the following formula [39]: summation ratio = 2nd measurement/1st measurement.

The only qualitative variable included was education level, which was divided into primary (1), secondary (2), and university (3) studies.

To prevent selection bias, we established a number of previously defined inclusion and exclusion criteria to decrease the differences within the study population. To prevent classification bias, the patients had to have a previous diagnosis from a medical specialist. Another bias of considerable importance could have been the consumption of medication. To avoid this bias, the scheduled patients were reminded not to consume any type of analgesic (except for those patients with preventive medication) in the 24 hours before the visit.

Finally, to address information bias, all the participants were provided with structured information on the study.

The sample size was calculated with the G.Power 3.1 program (University of Düsseldorf). According to our pilot study, we determined that a total of 50 participants divided into 3 groups were needed to obtain a minimum correlation of 0.4 (moderate) among the variables, accepting an alpha error of 0.05 and a statistical power of 90%.

We used SPSS version 2.0 for the data analysis and the Kolmogorov-Smirnov test for the normality tests. We employed the analysis of variance or the Kruskal-Wallis test to compare the means according to their distribution and Spearman's test to assess the correlation coefficient among the various variables for clinical sample. The results are presented with a 95% confidence interval (CI) for all the variables. The correlation coefficient was interpreted according to the values proposed by Cohen [40].

## 3. Results

A total of 59 female patients were recruited, with an average age of 44.88 ± 12.24 years (mean ± SD) with a weight of 64.93 ± 11.05 kg and a height of 1.64 ± 0.06 m (all of these values had a normal distribution).

According to the diagnoses, 20 patients had CM, 19 patients had TMD, and 20 were asymptomatic. One-way ANOVA showed no statistically significant differences in age, weight, and height between the groups (*p* > .05). All the values are shown in Table 1.

Regarding education level, 8.5% had primary-, 39% had secondary-, and 52.5% had university-level education. No statistically significant differences were found between the groups (*p* > .05).

### 3.1. Psychological Variables

The level of pain catastrophizing and kinesiophobia had a normal distribution.  Table 2(a) shows mean and standard deviations for both variables and pain catastrophizing subscales.

Statistically significant differences in pain catastrophizing were found when comparing the CM group with the asymptomatic group (mean difference [CI] 17.05 [6.8 to 27.3]; *p* = .000), its subscale rumination (CI 6.61 [2.8 to 10.4]; *p* = .000), and hopelessness (CI 8.63 [3.5 to 13.7]; *p* = .000). No statistically significant differences were found in magnification (*p* = .151) and kinesiophobia (*p* = .053) (Table 2(b)).

Statistically significant differences in pain catastrophizing were also found when comparing the TMD group with the asymptomatic group (CI 15.3 [5.1 to 25.5]; *p* = .002), including rumination (CI 5.4 [1.6 to 9.2]; *p* = .003), magnification (CI 2.67 [0.5 to 4.9]; *p* = .013), hopelessness (CI 7.2 [2.1 to 12.3]; *p* = .003), and kinesiophobia (CI 4.45 [0.4 to 8.5]; *p* = .026) (Table 2(b)).

No statistically significant differences were observed between the CM and TMD groups in pain catastrophizing and kinesiophobia.

### 3.2. Physical Variables

**Figure 1:**
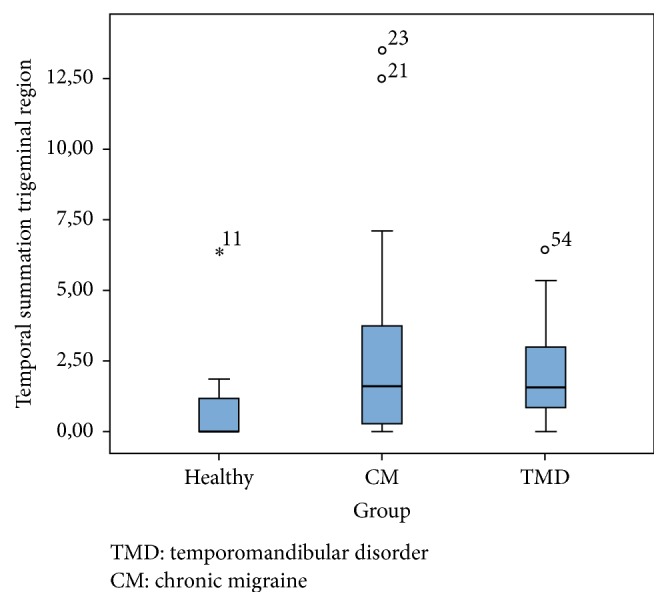
Boxplot for temporal summation in the trigeminal region.

**Figure 2:**
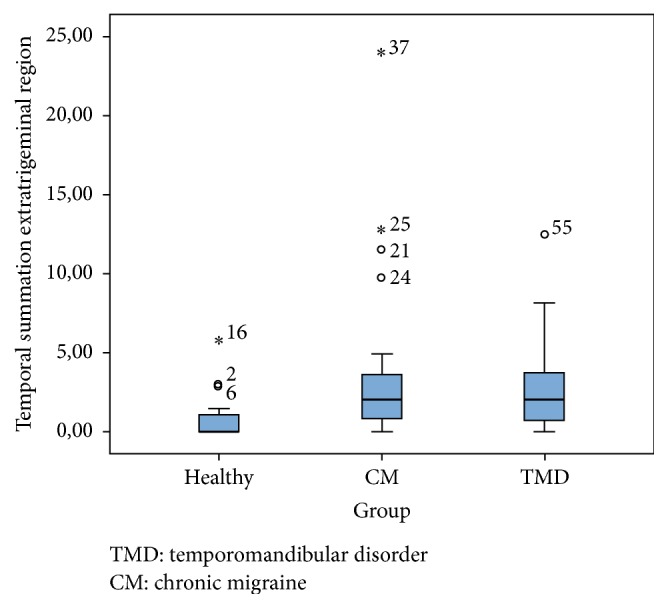
Boxplot for temporal summation in the extratrigeminal region.

The pain intensity, the magnitude of the temporal summation, and the educational level did not have normally distributed data. Statistically significant differences were observed in the Kruskal-Wallis test between both the CM and TMD groups with the asymptomatic group in pain intensity (*χ* ^2^ = 41.67; *p* = .000) and in magnitude of the temporal summation in the trigeminal area (*χ* ^2^ = 7.34; *p* = .02) (Figure 1) and in the extratrigeminal area (*χ* ^2^ = 10.64; *p* = .005) (Figure 2). No statistically significant differences were found between the CM and TMD groups in pain intensity (*χ* ^2^ = 2; *p* = .15) or the temporal summation in the trigeminal area (*χ* ^2^ = .008; *p* = .93) and extratrigeminal area (*χ* ^2^ = .006; *p* = .94).

### 3.3. Correlation Analyses for Clinical Sample

A Spearman correlation coefficient showed moderate and positive correlation between pain catastrophizing and kinesiophobia (*ρ* = 0.46; *p* = .001) for all the clinical samples. Chronicity had a moderate and positive correlation with catastrophizing (*ρ* = 0.45; *p* = .002) and kinesiophobia (*ρ* = 0.34; *p* = .01). Pain catastrophizing showed a moderate and positive correlation with pain intensity (*ρ* = 0.47; *p* = .001).

A strong correlation was observed in the temporal summation between the trigeminal and extratrigeminal areas (*ρ* = 0.64; *p* = .000). A very strong and positive correlation was found between both sides of the masseter in the temporal summation in the trigeminal area (*ρ* = 0.93; *p* < .01) and between both sides of the tibialis anterior in the temporal summation in the extratrigeminal area (*ρ* = 0.76; *p* < .01) (Table 3).

Finally, a strong positive correlation between chronicity and pain intensity was observed (*ρ* = 0.74; *p* = .00).

In the CM group, a moderate and positive correlation was observed between the magnification of pain and kinesiophobia (*ρ* = 0.54; *p* = .03). A moderate and negative correlation was found between chronicity and hopelessness (*ρ* = −0.51; *p* = .04).

No significant correlations were obtained in the TMD group.

## 4. Discussion

Chronic pain is one of the greatest challenges in pain management. As far as the authors know, this study is the first to compare groups of women with chronic CM or chronic TMD with healthy women. Our study showed differences between the CM group and the chronic TMD group compared with the group of asymptomatic participants for the variables of pain level catastrophizing, although these differences were not observed when comparing the CM group with the chronic TMD group. Differences in the level of kinesiophobia were only observed between the TMD group and the asymptomatic participants. In contrast, we observed no significant differences between the CM group and the asymptomatic group.

### 4.1. Catastrophizing and Kinesiophobia

According to these results, high levels of pain catastrophizing and kinesiophobia appear to be related to the presence or absence of chronic craniofacial pain, although they do not appear to be useful when characterizing either type of patient (CM or chronic TMD). Moreover, the correlation analysis revealed the presence of covariance between the levels of the 2 variables and the pain chronicity and intensity, such that the greater the catastrophism and/or kinesiophobia, the greater the pain chronicity and intensity. Previous studies have shown a positive moderate correlation between the level of pain catastrophizing and the pain intensity or severity in patients with different types of chronic pain [20, 21, 41]. Those studies and ours are consistent in terms of the approach to the pain chronicity condition described by Leeuw et al. in the fear-avoidance model [23]. According to this approach, patients with a high tendency to catastrophize pain can show higher levels of kinesiophobia, aggravating the fear of movement behavior by rumination, magnification, and hopelessness felt by individuals when facing their disease. There are numerous studies that have found positive moderate correlations between kinesiophobia and pain catastrophizing in patients with chronic pain such as CM, TMD, and fibromyalgia [36, 37, 42–44]. In contrast, Visscher et al. (2010) found a low correlation between kinesiophobia and pain catastrophizing in patients with TMD. The discrepancies between the findings of Visscher and those presented in this study could be due to the difference between the samples, given that theirs included 327 patients of both sexes [45]. However, when an analysis was performed for the factor group, the TMD group shows similar correlation results to the Visscher study between kinesiophobia and pain catastrophizing.

Differences in the level of kinesiophobia were only observed between the TMD group and the asymptomatic participants. This difference could be because the TMD diagnosis also included joint pain; therefore, a possible mechanical disorder could cause fear of movement. This situation does not occur in CH.

Finally, chronicity is associated with pain catastrophizing and kinesiophobia. Previously, other authors have noted that both pain catastrophizing and kinesiophobia can be predictors of chronic pain, especially when pain-related disability is present [46].

### 4.2. Magnitude of Temporal Summation and Pain Intensity

In this study, significant differences were observed when comparing the asymptomatic group with the CM and TMD groups. We observed no differences, however, between the latter 2 groups. As with our study, a previous study on patients with CM and episodic migraines showed significant differences compared with allodynia perceived in the various study groups [47].

The theory that associates chronic pain with supraspinal involvement is supported by a number of research studies of patients with chronic pain that found differences compared with the asymptomatic group using temporal summation tests, such as the pinprick stimuli test. A number of authors have observed generalized hyperalgesia in these patients beyond the area where the painful symptoms initially started, which could lead us to consider a central sensitization model [48]. Another research study performed by Maier et al. in 2010 with a larger sample than in the previous study confirmed that patients with nociceptive pain presented hyperalgesia and allodynia when performing various quantitative sensory tests. The study found a high percentage of patients with a diagnosis of trigeminal neuralgia in the sample [49].

In contrast, Raphael [50] found no significant differences in the temporal summation of painful temperature stimuli for women with a diagnosis of TMD compared with the controls. The authors found differences in the trigeminal nerve region (masseter) but not in the extratrigeminal regions (hands). These results could be due to the fact that there is sensitization in the area where the pain remains, which could not be compared with diseases such as fibromyalgia, in which general sensitization has been observed. Additionally and unlike our study, the participants of that study only included patients with a diagnosis of TMD of muscular origin, without specifying whether the TMD was chronic or not. Our sample, however, included patients with chronic TMD of muscle, joint and mixed origin. Another difference between the studies was the variability of race; Raphael included participants from other races, not just white, as in our study [50].

Although pain intensity showed differences between clinical groups (CM and chronic TMD) compared with asymptomatic participants, no differences were observed between the asymptomatic participants and the patients with CM or with chronic TMD. Some studies have already observed comorbidity between these two disorders [51]. Therefore, general pain intensity perception could be similar in both conditions.

### 4.3. Educational Level

No significant differences in terms of educational level among the groups were found in our study. In contrast, several previous studies conducted with patients with headaches showed a relationship between pain intensity and educational level. Chu et al. (2013) conducted a study on 1507 patients diagnosed with tension type headache (TTH) and migraine and showed that a university education was related to a lower prevalence of primary headaches in women [52]. The difference between their results and ours could be due to the differences in the participating population. The authors' inclusion criteria were men and women aged 19 to 69 years with a diagnosis of TTH or migraine, whereas our study included only women with a diagnosis of CM. Another possible reason for the lack of similarity in the results could have been the difference in sample size.

Two more research studies supporting the previous study revealed the negative correlation between educational level and the risk of developing migraines in women. The differences compared with our study could correspond to the fact that Le et al. had a sample composed of Danish men and women diagnosed with episodic migraine. In contrast, 100% of the sample from the study by Winter et al. were women diagnosed with different types of head pain, including headache [53, 54].

### 4.4. Clinical Implications

This study provides important information on the psychological factors of women with CM or with chronic TMD. These women showed higher levels of pain catastrophizing than asymptomatic subjects, which partially supports the applicability of the fear-avoidance model to these populations.

Chronicity has proven to be a factor associated with the presence of catastrophism and kinesiophobia; therefore, the prompt treatment of patients with pain might be useful to prevent the appearance of these two conditions. Developing strategies that combine psychological approaches to cognition or behavior and trying to modulate the pain perceived by patients with CM and chronic TMD are of great importance. Moreover, it is essential to consider the coping strategies for chronic craniofacial pain and their relationship with educational level. Drossman et al., in a study on patients with gastrointestinal disorders, showed a negative correlation between educational level and pain catastrophizing in terms of coping strategies [55]. Both the differences in the temporal summation of stimuli (when comparing the patient groups and the healthy group) and the strong relationship found bilaterally in the temporal summation of stimuli in the trigeminal and extratrigeminal areas support the possibility of developing a central sensitization process.

### 4.5. Limitations

This study has several limitations. One of the significant limitations of cross-sectional studies is the inability to establish causal relationships. The usefulness of this type of study, however, lies in creating new working hypotheses. Another limitation of our study is the possibility of potential sources of risk, such as the consumption of medication, despite the measures employed to avoid them. Regarding medication, medication overuse headache (MOH) has a high degree of comorbidity with CM. The psychological and physical aspects of these patients can differ from those of patients without MOH; thus the presence of this condition could have altered the results. The fact that data was lost in some of the questionnaires completed by the patients is also worth noting. The fact that the sample comes from a specific area (north of Madrid) could cause the results to vary when compared with other geographic areas. More than half of the sample had a higher than average education level. The relationship that this educational level could have with pain perception and on constructs such as fear of movement or pain catastrophizing has already been studied. A small part of the sample comes from an association of patients, which could involve especially motivated patients and those predisposed to collaboration. The data could not be extrapolated to men because this sample was composed exclusively of women. Thus, another limitation could be considered in regard to the patients' menstrual cycle phase. The authors believed that the groups were balanced in regard to fertility phase because there were no differences between the ages of the groups evaluated. However, the phase of the menstrual cycle in which they were at the time was not measured. Having been evaluated randomly, authors believe that the range of hormonal phases would be similar between groups, but this situation cannot be assured. Finally, another limitation is not having evaluated other psychosocial factors such as depression and anxiety, as well as their potential interactions, given that previous studies have observed an association with CM and TMD [18, 56].

### 4.6. Conclusion

In conclusion, no differences were observed in pain catastrophizing and kinesiophobia between women with CM and with chronic TMD. Women with CM or chronic TMD showed higher levels of pain catastrophizing than asymptomatic subjects. Only patients with chronic TMD had higher kinesiophobia than asymptomatic subjects.

## Figures and Tables

**Table 1:** Demographic characteristics for the sample.

	Age (years)	Weight (Kg)	Height (m)	BMI
Healthy	50.56 (11.95)	63.59 (7.03)	1.63 (0.073)	23.19 (2.56)
CM	38.33 (13.68)	66.26 (13.06)	1.65 (0.063)	24.11 (3.81)
TMD	48.10 (11.18)	65.10 (13.74)	1.64 (0.059)	24.08 (4.25)

CM: chronic migraine; TMD: temporomandibular disorder; BMI: body mass index.

All variables obtained a *p* value > .05.

**(a):** 

Group	Pain catastrophizing	Rumination	Magnification	Helplessness	Kinesiophobia
Healthy	8.82 (9.9)	3.82 (4.3)	1.82 (2.3)	3.18 (3.5)	19.24 (5.1)
CM	25.88 (11.9)	10.44 (4.1)	3.63 (2.2)	11.81 (6.8)	23.11 (4.4)
TMD	24.13 (13.4)	9.25 (4.6)	4.5 (3.1)	10.38 (6.8)	23.69 (4.4)
*p values*	*.00*	*.00*	*.014*	*.00*	*.016*

TMD: temporomandibular disorder; CM: chronic migraine.

**(b):** 

Variable	Comparison groups	Mean difference	CI 95%
Pain catastrophizing	Healthy, CM CM, TMD TMD, healthy	−17.05^*∗∗*^ 1.75 15.3^*∗∗*^	−27.3/−6.8 −8.65/12.15 25.55/5.05

Rumination	Healthy, CM CM, TMD TMD, healthy	−6.61^*∗∗*^ 1.18 5.42^*∗∗*^	−10.4/−2.83 −2.66/5.03 1.64/9.21

Magnification	Healthy, CM CM, TMD TMD, healthy	−1.8 −0.87 2.67^*∗*^	−4.03/0.42 −3.13/1.38 0.45/4.9

Helplessness	Healthy, CM CM, TMD TMD, healthy	−8.63^*∗∗*^ 1.43 7.19^*∗∗*^	−13.74/−3.53 −3.74/6.62 2.1/12.3

Kinesiophobia	Healthy, CM CM, TMD TMD, healthy	−3.87 −0.57 4.45^*∗*^	−7.79/0.04 −4.55/3.4 0.42/8.48

CI: 95% confidence intervals (lower limit/upper limit).

TMD: temporomandibular disorder; CM: chronic migraine.

^*∗*^ *p* < .05.

^*∗∗*^ *p* < .01.

**Table 3:** Spearman correlation coefficient between variables.

	Pain intensity	TSMD	TSMI	TSTD	TSTI	PCS	PCS (R)	PCS (M)	PCS (D)	TSK-11
Pain intensity	—									
TSMD	.307^*∗*^	—								
TSMI	.310^*∗*^	.934^*∗∗*^	—							
TSTD	.389^*∗∗*^	.585^*∗∗*^	.598^*∗∗*^	—						
TSTI	.292^*∗*^	.574^*∗∗*^	.577^*∗∗*^	.758^*∗∗*^	—					
PCS	.469^*∗∗*^	.336^*∗*^	.317^*∗*^	.256	.186	—				
PCS (R)	.455^*∗∗*^	.291^*∗*^	.263	.193	.156	.958^*∗∗*^	—			
PCS (M)	.334^*∗*^	.345^*∗*^	.327^*∗*^	.238	.120	.850^*∗∗*^	.791^*∗∗*^	—		
PCS (D)	.459^*∗∗*^	.351^*∗*^	.360^*∗*^	.289^*∗*^	.238	.959^*∗∗*^	.867^*∗∗*^	.785^*∗∗*^	—	
TSK-11	.266	.066	.105	.268	.206	.458^*∗∗*^	.460^*∗∗*^	.515^*∗∗*^	.434^*∗∗*^	—

TSMD: temporal summation right masseter; TSMI: temporal summation left masseter; TSTD: temporal summation right tibia; TSTI: temporal summation left tibia; PCS: pain catastrophizing scale; PCS (R): rumination; PCS (M): magnification; PCS (D): helplessness; TSK-11: Tampa Scale of Kinesiophobia.

^*∗*^ *p* < .05 (bilateral).

^*∗∗*^ *p* < .01 (bilateral).
